# Sequence specific suppression of androgen receptor–DNA binding *in vivo* by a Py-Im polyamide

**DOI:** 10.1093/nar/gkz153

**Published:** 2019-03-06

**Authors:** Alexis A Kurmis, Peter B Dervan

**Affiliations:** Division of Chemistry and Chemical Engineering, California Institute of Technology, Pasadena, CA, 91125, USA

## Abstract

The crucial role of androgen receptor (AR) in prostate cancer development is well documented, and its inhibition is a mainstay of prostate cancer treatment. Here, we analyze the perturbations to the AR cistrome caused by a minor groove binding molecule that is designed to target a sequence found in a subset of androgen response elements (ARE). We find treatment with this pyrrole-imidazole (Py-Im) polyamide exhibits sequence selectivity in its repression of AR binding *in vivo*. Differentially changed loci are enriched for sequences resembling ARE half-sites that match the Py-Im polyamide binding preferences determined *in vitro*. Comparatively, permutations of the ARE half-site bearing single or double mismatches to the Py-Im polyamide binding sequence are not enriched. This study confirms that the *in vivo* perturbation pattern caused by a sequence specific polyamide correlates with its *in vitro* binding preference genome-wide in an unbiased manner.

## INTRODUCTION

Transcription factors regulate cellular gene expression and the loss of this regulatory balance can lead to a myriad of genetic diseases including cancer. The role of androgen receptor (AR) in prostate cancer is one of the most well characterized examples. Early work in 1941 by Charles Huggins and Clarence Hodges showed that the progression of prostate cancer can be controlled by androgen deprivation through castration or hormonal therapy with estrogen (1). Later the discovery of the first anti-androgen, cyproterone acetate, allowed direct inhibition of androgen binding to the AR (2). Since then, the AR has remained the primary target for systemic therapeutics for prostate cancer patients (3,4). In recent years, newer anti-androgens including enzalutamide and apalutamide have already been approved and others are in late-stage clinical development (5–7).

Metastatic prostate cancers treated with androgen suppressive therapy will ultimately progress to a disease state termed castration-resistant prostate cancer (CRPC). Second-line AR directed therapeutics, such as enzalutamide, are often effective against CRPC, but a second disease progression is almost inevitable. Two mechanisms that have been documented to confer resistance to second-line AR directed therapies are mutations to the AR C-terminal ligand-binding domain and expression of AR splice variants lacking the ligand-binding domain (8–10). Multiple approaches have been explored to overcome these resistance mechanisms, as reviewed recently by Jung *et al.* (11). These include AR transcription activation domain inhibitors such as EPI-506 and AR DNA-binding domain inhibitors, such as pyrvinium pamoate (11). In addition, our lab has previously reported the use of DNA binders to allosterically modulate the binding of AR at the protein–DNA interface (12). We have shown this approach to be efficacious in several prostate cancer models, including anti-androgen resistant models (13,14).

Pyrrole-imidazole (Py-Im) polyamides are DNA minor groove binding molecules with modular sequence specificity that bind to target sites with affinities comparable to DNA-binding proteins (15,16). Minor groove sequence recognition is determined by the pairing of N-methylimidazole (Im) and N-methylpyrrole (Py); the target sequence of a particular polyamide is dependent on the location of the Im and Py monomers within the hairpin structure (17). An Im/Py pair will recognize a G•C pair in the DNA, Py/Im will recognize C•G and Py/Py will bind to either A•T or T•A (18–20). Upon binding to the minor groove, Py-Im polyamides cause an expansion of the minor groove and a corresponding compression in the opposing major groove (21). Py-Im polyamides have been shown to interfere with DNA dependent processes such as gene expression, RNA polymerase II elongation, DNA polymerase replication and topoisomerase activity (13,22–24). They have also been shown to activate p53 and induce apoptosis without genotoxicity, and to have antitumor activity in prostate cancer cell lines and xenograft models (13,14,23). **ARE-1** is a Py-Im polyamide designed to target the sequence 5′-WGWWCW-3′, where W represents either A or T, which is found in a subset of androgen response elements (ARE).

In this study, we evaluate the anti-proliferative effects of **ARE-1** in the setting of enzalutamide resistant LNCaP-95 cells, and in the context of AR signaling. We further examine the disruption pattern to the cistrome caused by **ARE-1** treatment. We find that at loci where AR binding is reduced by **ARE-1** treatment, the consensus ARE motif bears closer resemblance to the **ARE-1** target sequence, whereas the native consensus motif has more sequence degeneracy.

## MATERIALS AND METHODS

### Cell culture

The LNCaP-95 cell line was obtained from the laboratory of Dr. Jun Luo at Johns Hopkins School of Medicine. The cells were received at passage 3 and maintained in phenol red free RPMI 1640 (Gibco 11835-030) with 10% charcoal treated fetal bovine serum (CTFBS). All experiments were performed below passage 20, and cells were validated to parental cell line and confirmed mycoplasma free by ATCC following experimentation.

### Cell uptake

Cell uptake was confirmed by confocal imaging. Briefly, LNCaP-95 cells were plated in 35-mm optical dishes (MatTek) at 7.5 × 10^4^ cells per dish and allowed to adhere for 24 h. Cells were treated with 2 μM **ARE-1-FITC** for 16 h, washed with phosphate buffered saline (PBS) and imaged at the Caltech Biological Imaging Facility using a Zeiss LSM 710 inverted laser scanning confocal microscope equipped with a 63× oil immersion lens.

### Cytotoxicity assay

LNCaP-95 cells were plated at 7.5 × 10^3^ per well in 96 well plates. Cells were allowed to adhere for 24 h, and media was then replaced with fresh media containing vehicle or polyamide **ARE-1**. After 72 h, an equivalent volume of CellTiter-Glo (CTG) reagent (Promega) was added to each well. Luminescence was allowed to stabilize for 10 min at room temperature, according to manufacturer instructions, and then measured on a FlexStation3 plate reader (Molecular Devices). Background subtracted luminescence of polyamide treated cells was normalized to vehicle treated cells, and non-linear regression analysis (Prism software, Graphpad) was performed to determine IC_50_ value.

### Gene expression analysis by quantitative RT-PCR (qPCR)

LNCaP-95 cells were cultured for 24 h after plating in six well plates at 7.5 × 10^4^ cells/ml. Cells were treated with 10 μM **ARE-1** with 10 nM dihydrotestosterone (DHT) or DMSO for 24 h before harvest. RNA extraction (RNEasy columns, Qiagen), complementary DNA (cDNA) generation (ProtoScript II First Strand cDNA Synthesis Kit, NEB), and qRT-PCR (PowerUp SYBR Green Master Mix, Life Technologies, ABI7300 instrument) were done following manufacturer recommendations. Expression was normalized to β-glucuronidase.

### Bioavailability in new formulation

All animal experiments were performed at the California Institute of Technology (Pasadena, CA) with prior IACUC approval. To evaluate a new formulation for polyamide delivery, **ARE-1** was injected at 10 mg/kg in a 1% polyvinylpyrrolidone 10 (PVP), 50 mM Tris, 0.9% saline vehicle into the right flank of 6 C57BL/6J mice. Mice were anesthetized using isoflurane and blood collected retro-orbitally at 30 min, 1, 3, 6, 12 and 24 h after injection. Blood samples were centrifuged at 6000 rpm for 5 min to collect the serum, which was processed as previously published and analyzed by HPLC to determine polyamide concentration (25). 9-aminoacridine was used as an internal standard.

### Xenograft assay

Male SCID hairless outbred mice (4–6 weeks old) were obtained from Charles River Laboratories. LNCaP-95 cells (3 × 10^6^) were injected into the flanks of the mice as a 1:1 mixture in Matrigel (BD Biosciences). Mice were monitored for the appearance of tumors and calipered twice weekly once tumors appeared. When tumors reached 100 mm^3^ (using 0.5*l*w*w), animals were castrated by veterinary staff. Following surgery, animals were monitored daily for 3 days, and allowed to recover for 7–10 days prior to the start of treatment. After the recovery period, animals were randomly assigned to treated or vehicle groups, and injected three times per week with 2.5 mg/kg **ARE-1** or vehicle (1% polyvinylpyrrolide 10 (PVP), 50 mM Tris, 0.9% saline) for 3 weeks. Tumor growth was monitored weekly by calipers, and growth compared to starting size. Animals were anesthetized with 2–5% isoflurane/air when necessary, and sterile technique was used for all procedures. Animal health was monitored daily by veterinary staff, and any animals exhibiting signs of distress were euthanized by administration of isoflurane followed by carbon dioxide.

### Chromatin immunoprecipitation

Genomic occupancy of full-length AR was determined by chromatin immunoprecipitation (ChIP) with the PG21 AR antibody (Millipore). LNCaP-95 cells were plated at 20 million cells per plate in phenol red-free RPMI 1640 supplemented with 10% CTFBS and allowed to adhere for 24 h. The cells were treated with 10 μM **ARE-1** with either 10 nM DHT or DMSO for 24 h. Crosslinking was performed with 1% formaldehyde in media for 15 min followed by quenching with 0.125 M glycine. The cells were then washed with ice-cold PBS twice and harvested. Chromatin was sheared by sonication at −20°C at 25% amplitude in 30 s on and 10 s off cycles for 30 cycles. Next, 1 mg of sheared chromatin was incubated with PG21 antibody that was previously immobilized on Dynabeads (Invitrogen) overnight at 4°C. Samples were then washed 5× with LiCl buffer (10 mM Tris, 500 mM LiCl, 1% NP-40, 1% sodium deoxycholate) and once with TE buffer. DNA was then harvested by phenol chloroform extraction and purified using the Monarch PCR & DNA Cleanup kit (NEB). Quantitative polymerase chain reaction (qPCR) was used to validate enrichment at the *KLK3* ARE I site (5′-TGCATCCAGGGTGATCTAGT-3′ and 5′-ACCCAGAGCTGTGGAAGG-3′) compared to a negative internal locus (5′-TAGAAGGGGGATAGGGGAAC-3′ and 5′-CCAGAAAACTGGCTCCTTCTT-3′) prior to submission for sequencing. Each sample was immunoprecipitated as three technical replicates, which were combined for sequencing on an Illumina HiSeq2500. Biological replicates of each treatment condition were acquired. Input DNA (not immunoprecipitated) was also extracted and purified using the same methods and submitted for sequencing.

### ChIP-Seq analysis

At least 29.7 million reads were sequenced for each sample. Reads were mapped to the human genome (hg19) using Bowtie2 v 2.2.3 and converted to BAM format with SAMtools (26,27). Peak calling was performed using the model-based analysis of ChIP-Seq (MACS2) program for each replicate (28). Peaks from each replicate of each condition were compared using irreproducible discovery rate (IDR) to determine a set of reproducible peaks, which was then submitted to multiple EM for motif elicitation (MEME)-ChIP (http://meme-suite.org/tools/meme-chip) for motif analysis (29–31). Peaks selected by IDR were converted to bigWig format for viewing in the UCSC genome browser (http://genome.ucsc.edu).

Differential analysis between treatment conditions was conducted using peak-calling prioritization (PePr) with a *P*-value cutoff of 1 × 10^−5^, sharp peaks and intra-group normalization (32). PePr results were used for all further analysis. BEDtools was used for overlap analysis and peak annotation was performed using ChIPseeker (33,34). Differentially changed peaks were submitted to MEME-ChIP for motif finding as above. Based on the MEME-ChIP results, Homer was used to examine the density of specific motifs within peaks (35). Data has been deposited and can be accessed in GEO (GSE125552).

### Thermal stabilization assay

Melting temperature analysis of the DNA oligos 5′-TTGTAGAACACGTT-3′, 5′-TTGTAGGACACGTT-3′, 5′-TTGTGGAACACGTT-3′ and 5′-TTGTGGGACACGTT-3′ in the presence of **ARE-1** was conducted as previously described (36).

### Statistical analysis

All statistical analysis was performed in GraphPad Prism. Gene expression data were normalized to the DHT induced condition and ANOVA analysis was performed on three biological replicates using the Dunnett’s test for multiple comparisons. Statistical analysis of tumor percentage growth between vehicle and **ARE-1** treated groups (*N* = 11 per group) was performed using the unpaired *t*-test. All reported *P*-values are two-sided.

## RESULTS

### Nuclear uptake and cytotoxicity

**Figure 1.**
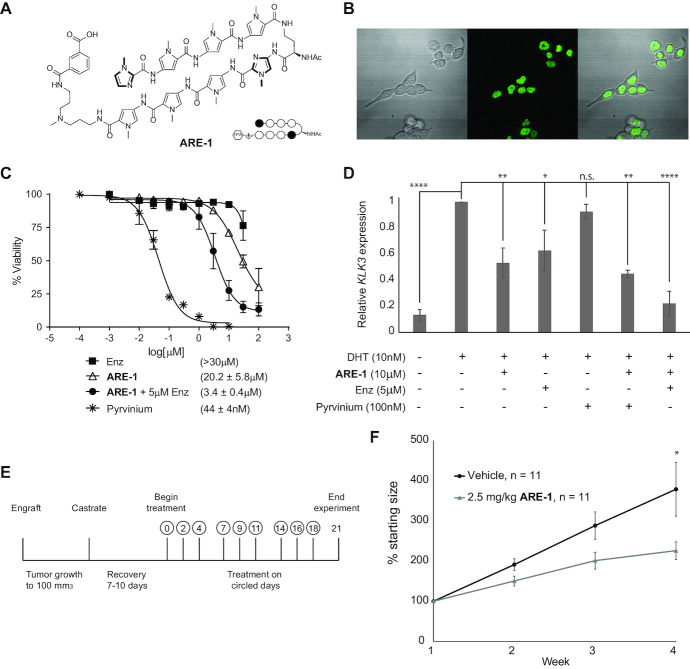
(**A**) Structure of Py-Im polyamide **ARE-1**. (**B**) Nuclear localization of **ARE-1-FITC** in LNCaP-95 cells after 16-h treatment. (**C**) Cell viability as determined by CellTiterGlo assay after 72-h treatment. IC_50_ is indicated in parentheses. (**D**) Relative expression of *KLK3* in LNCaP-95 cells after the indicated treatments. Cells were co-treated with **ARE-1**, enzalutamide, or pyrvinium pamoate and DHT at the indicated concentrations and RNA harvested at 24 h. (**E**) Schedule of LNCaP-95 xenograft experiment. Animals were engrafted, allowed to grow tumors, castrated, allowed to recover for 7–10 days and then treated with **ARE-1** or vehicle at indicated times. (**F**) Tumor volumes of LNCaP-95 xenografts in castrated mice treated with vehicle or 2.5 mg/kg **ARE-1** as shown in E. *N* = 11 for both groups; * *P* < 0.05, ** *P* < 0.01, **** *P* < 0.0001.

Py-Im polyamide **ARE-1** has been previously shown to exhibit antiproliferative activity toward several models of prostate cancer including LNCaP, LNCaP-AR, VCaP and LREX′ (14,22). We further evaluate the activity of **ARE-1** in LNCaP-95 cells, which derive their resistance from the expression of AR splice variants (37). Nuclear localization of **ARE-1** (Figure 1A) was confirmed using a fluorescein analog, **ARE-1-FITC** (Supplementary Figure S1), in LNCaP-95 cells (Figure 1B). Antiproliferative effect of **ARE-1** toward LNCaP-95 cell growth was evaluated using the CTG assay and compared against the antiandrogen enzalutamide and pyrvinium pamoate (pyrvinium), a molecule that has been reported to bind to the AR DNA-binding domain to prevent AR–DNA interactions (38). Results from the assay show the 72 h growth inhibition IC_50_s for **ARE-1**, enzalutamide and pyrvinium to be 20.1 μM, >30 μM and 44 nM, respectively. A synergistic effect was observed when a subtoxic concentration of enzalutamide (5 μM) was combined with polyamide, and the IC_50_ was reduced to 3.4 μM. Changes to *KLK3* gene expression was also evaluated in LNCaP-95 cells treated with **ARE-1**, enzalutamide, pyrvinium, and a combination of **ARE-1** with pyrvinium or enzalutamide (Figure 1D). After 24 h of treatment, the greatest reduction in *KLK3* expression from treatment with a single agent came from **ARE-1**, and combining either additional agent with **ARE-1** further reduced gene expression. Based on these cell culture results, we further evaluated the antitumor effects of **ARE-1** in LNCaP-95 xenografts using an optimized formulation that increased the subcutaneous bioavailability when compared to the previously used DMSO/saline vehicle (Supplementary Figure S2A). Animals were engrafted with LNCaP-95 cells and monitored until palpable tumors were observed. Once tumors reached 100 mm^3^, the animals were castrated, allowed to recover for ∼1 week and then randomized before treatment (Figure 1E). The animals were treated with either vehicle or 2.5 mg/kg **ARE-1** subcutaneously Monday/Wednesday/Friday for 3 weeks. The vehicle treated group grew ∼380%, while the **ARE-1** treated group grew 225%, for a 40% reduction in tumor size in the polyamide treated mice (Figure 1F). Animal weight was measured at each injection and was not adversely affected (Supplementary Figure S2B).

### Genomic perturbation of androgen receptor occupancy

**Figure 2.**
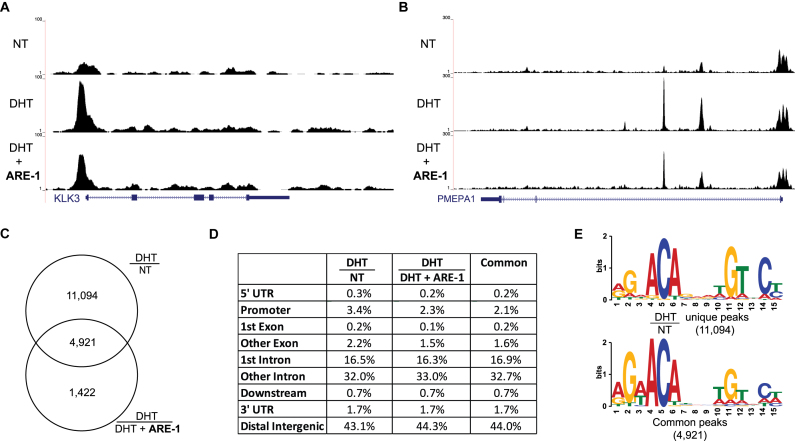
ChIP-seq analysis of the *KLK3* promoter ARE I (**A**) and *PMEPA1* (**B**). (**C**) Overlap of peaks differentially changed in DHT relative to NT, and peaks changed in DHT relative to DHT and **ARE-1**. (**D**) Identified peaks were annotated by genomic region by ChIPseeker. (**E**) Top ARE motif found by MEME in the indicated peak sets.

The effects of Py-Im polyamide treatment on AR occupancy on chromatin have previously been explored by ChIP experiments. A related Py-Im polyamide, targeting the same sequence as **ARE-1**, has previously been shown to decrease occupancy of AR at the *KLK3* promoter and enhancer in LNCaP cells (12). In LNCaP-95 cells, a similar reduction at the *KLK3* promoter ARE I is seen after 24 h of co-treatment with **ARE-1** and 10 nM DHT (Supplementary Figures S3A). In this study, we explored the genomic effect **ARE-1** treatment has on AR occupancy using ChIP-Seq analysis. Sequencing results of biological duplicates of non-treated (NT), 10 nM DHT treated (DHT), and 10 nM DHT and 10 μM **ARE-1** treated (DHT+**ARE-1**) showed ∼30 million reads mapping for all samples (Supplementary Figure S3B). Sequencing reads were aligned to hg19 and select AR target genes are shown (Figure 2A and B). Motif analysis by MEME discovered the forkhead-binding motif in all samples, and the complete ARE was discovered in the DHT and DHT + **ARE-1** samples (Supplementary Figure S3C). Differential binding of DHT/NT and DHT/(DHT+**ARE-1**) was calculated using PePr. Analysis revealed 16,015 peaks increased in DHT over non-treated (DHT/NT) and 6343 differentially changed DHT/(DHT+**ARE-1**) peaks, of which 4921 overlapped with DHT inducible peaks (Figure 2C). Correlation of peak location to genomic regions, conducted by ChIPseeker, showed no difference between the DHT/NT, DHT/(DHT+**ARE-1**), and overlap peaks, suggesting that **ARE-1** does not have a regional binding preference (Figure 2D). Motif analysis of peaks unique to DHT/NT revealed the canonical ARE where the first half-site is 5′-RGNACA-3′. In this motif, the first position is selective for A or G (R) and the third position is degenerate for any base (N) (Figure 2E). Motif analysis of the overlapping peaks between DHT/NT and DHT/(DHT+**ARE-1**) also revealed a complete ARE, however the first half-site has the sequence 5′-RGWACA-3′, where the third position shows selectivity for A or T (Figure 2E); additional motifs can be found in Supplementary Figure S4A. Comparison of the letter probability matrix between the DHT/NT unique peaks and the overlapping peaks show more A character in the first position and reduced C and G character in the third position in the overlapping motif (Supplementary Figure S4B).

**Figure 3.**
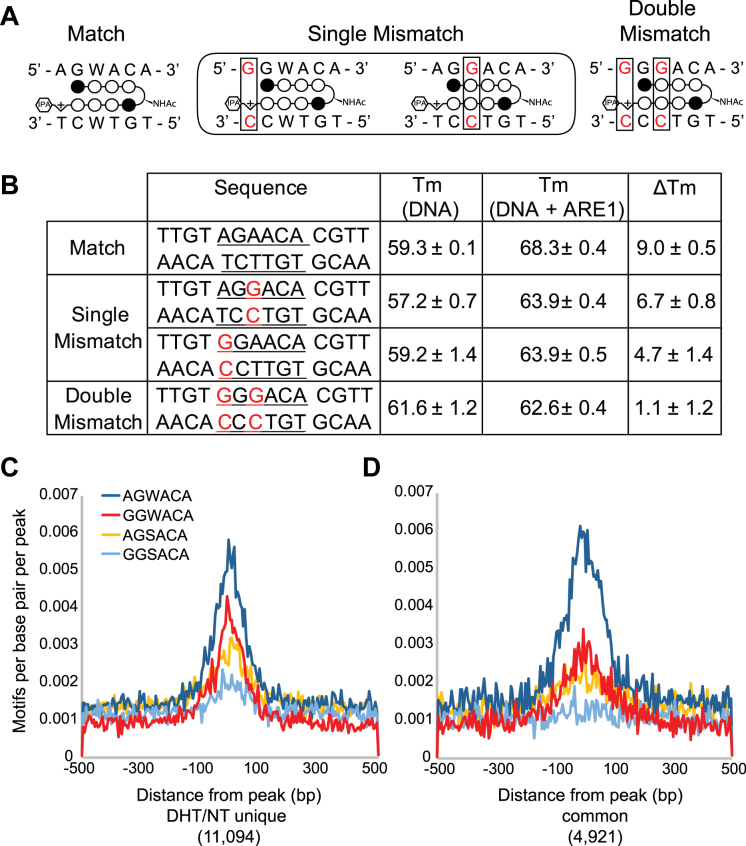
(**A**) Illustration of match and mismatch **ARE-1** binding sites. Mismatches are shown in red and boxed. (**B**) Melting temperature analysis of match and mismatch sequences shown in A. C + D) Motif density analysis of possible ARE half sites in peaks unique to DHT/NT (**C**) and overlapping between DHT/NT and DHT/DHT + **ARE-1** (**D**).

Of the possible permutations of the first ARE half-site, **ARE-1** is expected to have the strongest binding to the sequences 5′-AGWACA-3′. Based on Py-Im polyamide pairing rules, **ARE-1** is expected to have lower binding to the sequences 5′-GGWACA-3′ and 5′-AGGACA-3′, which contain single base mismatches, and to have little binding to the sequence 5′-GGGACA-3′, which contains two mismatches (Figure 3A) (17–20). DNA thermal stability experiments confirmed this trend and showed that **ARE-1** stabilized match sequences by ∼9°C; single mismatches reduced thermal stability by ∼2–4°C. **ARE-1** showed no significant thermal stabilization to a double mismatch sequence (Figure 3B).

The ARE half-site sequence 5′-RGNACA-3′ can be split into four sequences: 5′-AGWACA-3′, 5′-GGWACA-3′, 5′-AGSACA-3′ and 5′-GGSACA-3′, where S represents G or C. Density analysis of these four motifs revealed 5′-AGWACA-3′ to be significantly enriched around the peak center of DHT/NT and DHT/(DHT+**ARE-1**) overlap peaks compared to the other possible motifs. A lesser effect was found for the DHT/NT unique peaks (Figure 3C–D).

**Figure 4.**
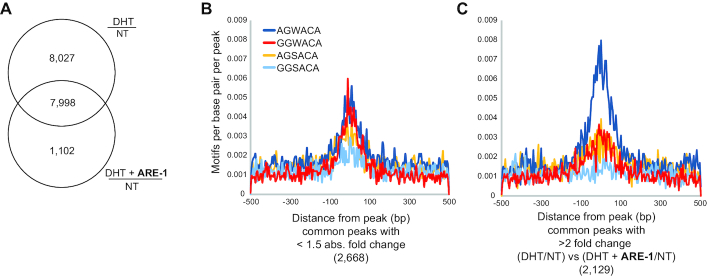
(**A**) Venn diagram showing the overlap of peaks differentially changed in DHT as compared to NT with peaks changed by DHT and **ARE-1** as compared to NT. (**B** and **C**) Density analysis of overlapping peaks with <1.5 absolute fold change between DHT/NT and DHT+**ARE-1**/NT (B) and peaks with >2-fold change when comparing DHT/NT to DHT+**ARE-1**/NT (C).

To confirm that the enrichment for 5′-AGWACA-3′ was only present in regions where AR peaks are affected by **ARE-1**, we examined common peaks between DHT/NT and (DHT+**ARE-1**)/NT samples (Figure 4A). Of the 7998 overlapping peaks, 2668 peaks had an absolute change of <1.5-fold. Motif density analysis in these unchanged regions showed no enrichment of 5′-AGWACA-3′ (Figure 4B). Comparatively, 5′-AGWACA-3′ was significantly enriched in 2129 peaks showing >2-fold change between DHT/NT and (DHT+**ARE-1**)/NT.

## DISCUSSION

Py-Im polyamides have been shown to inhibit the signaling of oncogenic transcription factors and reduce their binding at select loci in ChIP experiments (12,39,40). Genomic binding of Py-Im polyamides linked to DNA alkylators have also been examined (41,42). In this study, we elucidate the genome-wide effects of polyamide treatment on the AR on chromatin. Py-Im polyamide **ARE-1** is a cell permeable molecule that exerts anti-proliferative effects toward several prostate cancer models, including the castration and enzalutamide resistant models LREX’ and now LNCaP-95.

In this present study, we find that **ARE-1** localizes to LNCaP-95 nucleus within 16 h of dosing, and is able to repress ligand-induced gene expression after 24 h of co-treatment with DHT. In this time frame, our ChIP-Seq results show **ARE-1** is able to repress ∼30% of DHT inducible peaks. Motif analysis of these AR peaks repressed by **ARE-1**, which is selective for the sequence 5′-WGWWCW-3′, indicate that these loci are enriched for canonical AREs with 5′-RGWACA-3′ as the first half-site compared to the common 5′-RGNACA-3′ half-site. Thus, the differential effects on AR-DNA binding events *in vivo* reflects the DNA target sequence binding preference of **ARE-1***in vitro*. These experiments provide evidence of the *in vivo* sequence selectivity of **ARE-1**, and provide a snapshot of how **ARE-1** modulates the AR cistrome.

## DATA AVAILABILITY

Data deposited in GEO: series GSE125552

## Supplementary Material

Supplementary Data
