# Supplementary material for: Sequence specific suppression of androgen receptor–DNA binding in vivo by a Py-Im polyamide
Source: Nucleic Acids Res. 2019 Mar 6;47(8):3828–35. doi: 10.1093/nar/gkz153 (PMC6486638; doi:10.1093/nar/gkz153)
Supplement: Supplementary Data [file gkz153_supplemental_file.pdf]

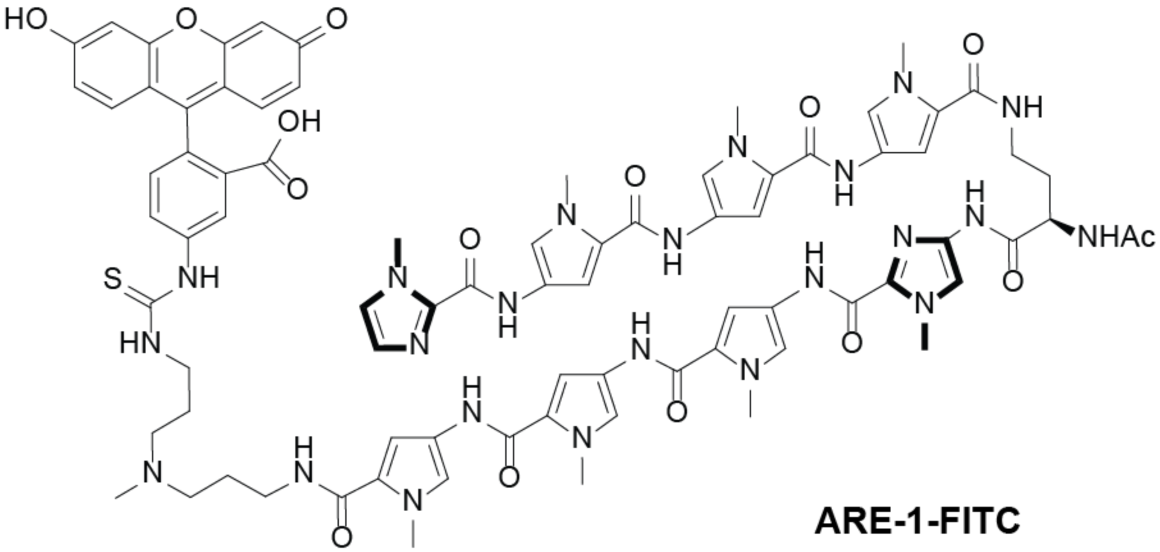

Figure S1. Chemical structure of **ARE-1-FITC**

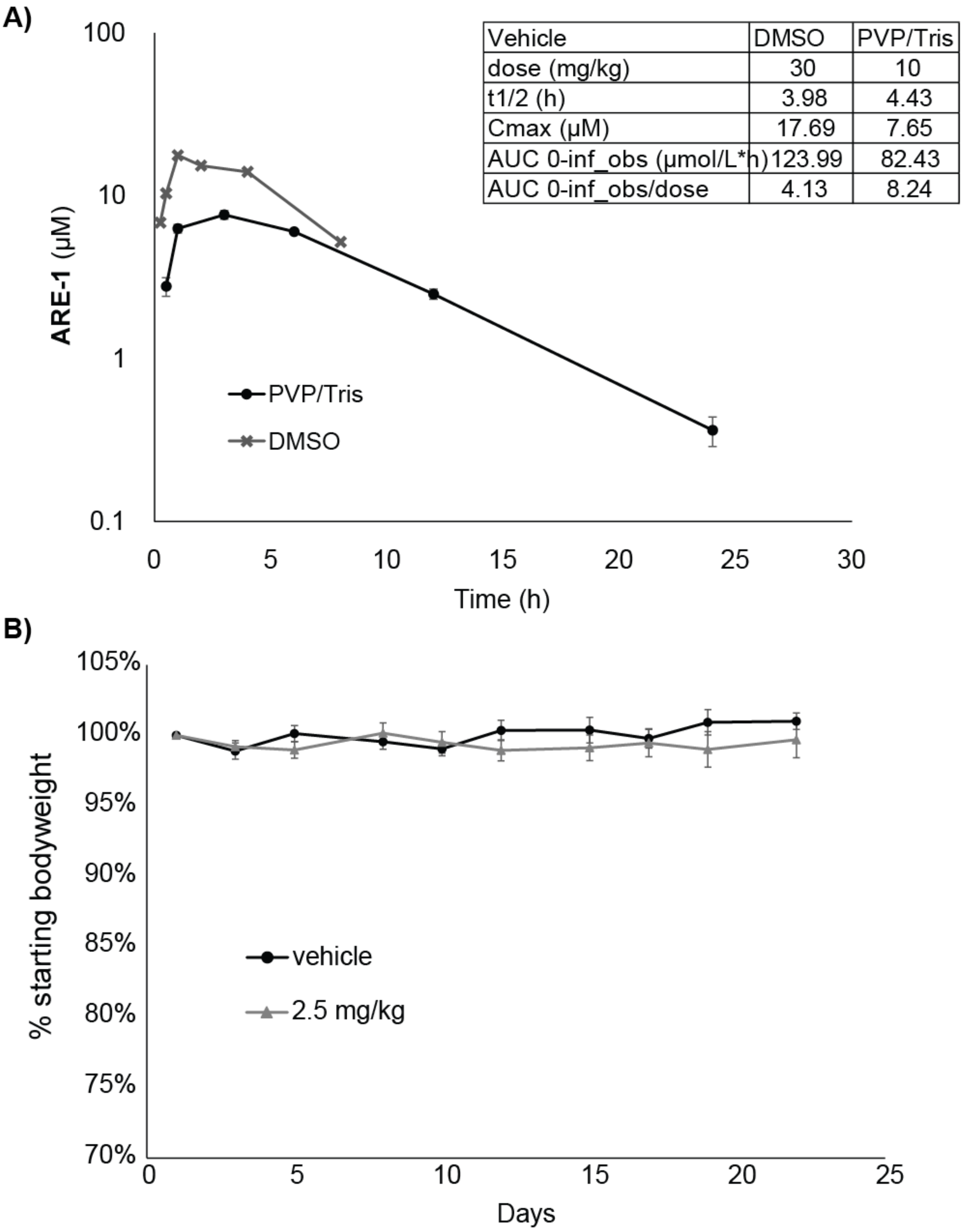

Figure S2. A) Pharmacokinetic analysis of **ARE-1** in PVP/Tris formulation compared to DMSO/saline formulation. **B)** Body weight changes of animals in LN95 xenograft experiment.

A)

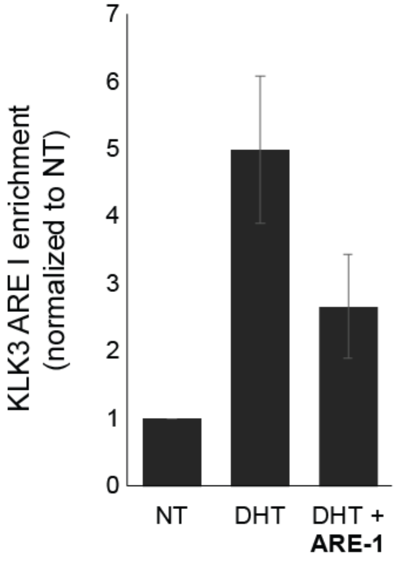

B)

	NT	DHT	DHT + **ARE-1**
Reads mapped (millions):			
rep 1	30.2	29.7	33.1
rep 2	30.0	43.5	44.5
(30 million recommended)			
Peaks:			
IDR	14,812	50,445	35,856
(rep 1)	(35,723)	(62,770)	(55,009)
(rep 2)	(24,801)	(84,015)	(45,102)

C)

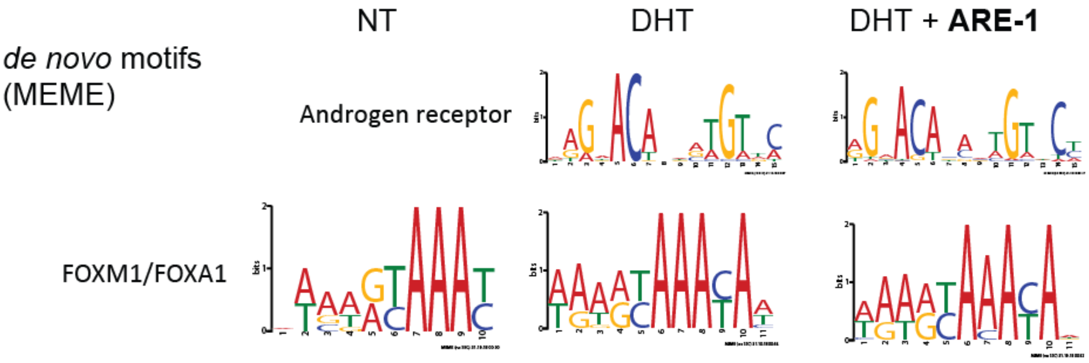

Figure S3. A) AR occupany of KLK3 ARE I as determined by ChIP. **B)** Sequencing results from AR ChIP experiments of indicated conditions. **C)** *De novo* motif discovery by MEME of reproducible peaks as determined by IDR analysis.

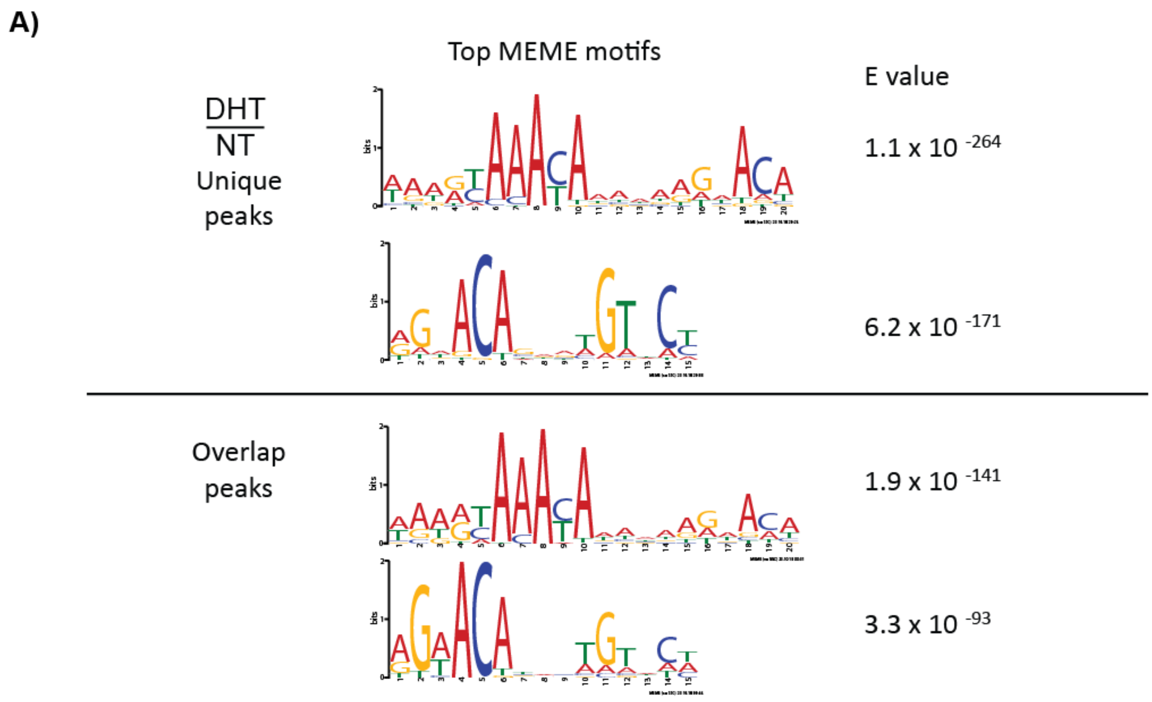

B)

DHT/NT unique A	DHT/NT unique C	DHT/NT unique G	DHT/NT unique T	Overlap A	Overlap C	Overlap G	Overlap T
0.426237	0.000000	0.405094	0.168669	**0.632749**	0.000000	**0.270216**	0.097035
0.137914	0.017780	0.756367	0.087938	0.000000	0.000000	0.923854	0.076146
0.398606	0.093224	0.192456	0.315714	**0.578841**	0.057278	**0.000000**	0.363881
0.894762	0.005766	0.068957	0.030514	1.000000	0.000000	0.000000	0.000000
0.000000	0.974291	0.025709	0.000000	0.000000	1.000000	0.000000	0.000000
0.913023	0.003364	0.002643	0.080971	0.884097	0.000000	0.027628	0.088275
0.142960	0.227775	0.481019	0.148246	0.134097	0.237871	0.216981	0.411051
0.414224	0.143681	0.253484	0.188611	0.333558	0.201482	0.165094	0.299865
0.420471	0.140317	0.302739	0.136473	0.281671	0.327493	0.145553	0.245283
0.265738	0.160500	0.028111	0.545651	0.274259	0.062668	0.047170	0.615903
0.063671	0.010332	0.925997	0.000000	0.125337	0.066712	0.807951	0.000000
0.135031	0.035560	0.032436	0.796973	0.210916	0.099057	0.068059	0.621968
0.225613	0.181643	0.194858	0.397886	0.377358	0.194744	0.152965	0.274933
0.120855	0.849351	0.000000	0.029793	0.195418	0.645553	0.000000	0.159030
0.135992	0.364969	0.007448	0.491591	0.371968	0.229111	0.000000	0.398922

Figure S4. A) Motif analysis of differentially bound sites using MEME-ChIP. Top two results are shown for peaks unique to DHT/NT and overlapping betwen DHT/NT and DHT/DHTP. **B)** Motif matrix of Androgen response element motif logo presented in **(A).** Positions 1 and 3 are highlighted in red.
